# Non-fusion palliative spine surgery without reconstruction is safe and effective in spinal metastasis patients: retrospective study

**DOI:** 10.1038/s41598-021-97056-2

**Published:** 2021-09-01

**Authors:** Siravich Suvithayasiri, Borriwat Santipas, Sirichai Wilartratsami, Monchai Ruangchainikom, Panya Luksanapruksa

**Affiliations:** 1 Orthopedic Center, Chulabhorn Hospital, HRH Princess Chulabhorn College of Medical Science, Chulabhorn Royal Academy, Bangkok, Thailand; 2 Department of Orthopedic Surgery, Faculty of Medicine, Siriraj Hospital, Mahidol University, 2 Wanglang Road, Bangkoknoi, Bangkok, 10700 Thailand

**Keywords:** Health care, Neurology

## Abstract

Considering the shorter life expectancy and poorer prognosis of metastatic epidural spinal cord compression patients, anterior reconstruction and fusion may be unnecessary. This study aimed to investigate the outcomes of palliative surgery for metastatic epidural spinal cord compression with neurological deficit among patients who underwent posterior decompression and instrumentation without fusion or anterior reconstruction. This single-center retrospective review included all patients aged > 18 years with thoracic or lumbar spinal metastasis who were surgically treated for metastatic spinal cord compression without fusion or anterior reconstruction at the Department of Orthopaedic Surgery, Faculty of Medicine Siriraj Hospital, Mahidol University, Bangkok, Thailand during July 2015 to December 2017. Data from preoperation to the 1-year follow-up, including demographic and clinical data, Frankel classification, pain scores, complication, revision surgery, health-related quality-of-life scores, and survival data, were collected and analyzed. A total of 30 patients were included. The mean age was 59.83 ± 11.73 years, and 20 (66.7%) patients were female. The mean operative time was 208.17 ± 58.41 min. At least one Frankel grade improvement was reported in 53.33% of patients. The pain visual analog scale, the EuroQOL five-dimension five-level utility score, and the Oswestry Disability Index were all significantly improved at a minimum of 3 months after surgery. No intraoperative mortality or instrument-related complication was reported. The mean survival duration was 11.4 ± 8.97 months. Palliative non-fusion surgery without anterior reconstruction may be considered as a preferable choice for treating spinal metastasis patients with spinal cord compression with neurological deficits.

## Introduction

Spinal metastasis is a known cause of severe pain and neurological deficit, and these effects adversely affect patient quality of life. Combination treatment of surgery and radiation was superior to radiation alone in patients with spinal metastasis relative to ambulation status and the need for analgesia^1^. Decompression via a posterior approach with or without instrumentation was reported to be a preferable surgical strategy^2^. Furthermore, many studies found good to excellent outcomes with the addition of anterior column reconstruction and fusion^3–9^. However, this patient population has a shorter life expectancy and a poorer prognosis, so it has been argued that these add-on procedures may unnecessarily increase operative time, intraoperative blood loss, and postoperative morbidity^10,11^.

This study aimed to investigate the outcomes of palliative surgery for metastatic epidural spinal cord compression with neurological deficit among patients who underwent posterior decompression and instrumentation without fusion or anterior reconstruction. We hypothesized that postoperative health-related quality-of-life (HRQoL) scores would be significantly improved.

## Material and methods

This single-center retrospective review included all patients aged > 18 years with thoracic (T1–T10), thoracolumbar (T11–L1), or lumbar (L2–L5) spinal metastasis who underwent posterior decompression and instrumentation without fusion or anterior reconstruction for symptomatic metastatic spinal cord compression with the neurological deficit at the Department of Orthopaedic Surgery, Faculty of Medicine Siriraj Hospital, Mahidol University, Bangkok, Thailand during July 2015 to December 2017. Patients with unknown primary cancer origin, who underwent fusion, and/or who received anterior reconstruction were excluded. Patient demographic and clinical data, including age, gender, primary cancer origin, and spinal region involvement, were collected. The patient's neurological status was assessed using the Frankel grading system. The health-related quality-of-life (HRQoL) was evaluated using the pain visual analog scale (VAS), the Oswestry Disability Index (ODI), and the EuroQOL five-dimension five-level (EQ5D5L) utility score. These assessment parameters were collected and compared between the pre-operative period and the 3-month, 6-month, and 12-month postoperative follow-ups. Surgery-related complication data, operative time, estimated blood loss, and length of hospital stay was also recorded and analyzed. Overall survival was calculated from the date of surgery to the date of final follow-up or death.

The Institutional Review Board (IRB) of the Faculty of Medicine Siriraj Hospital approved this study and approved for an exemption of informed consent, certificate of approval 833/2557(EC2). All the patient profiled was concealed by using the case number instead. All methods were performed following the relevant guidelines and regulations.

### Surgical technique

**Figure 1:**
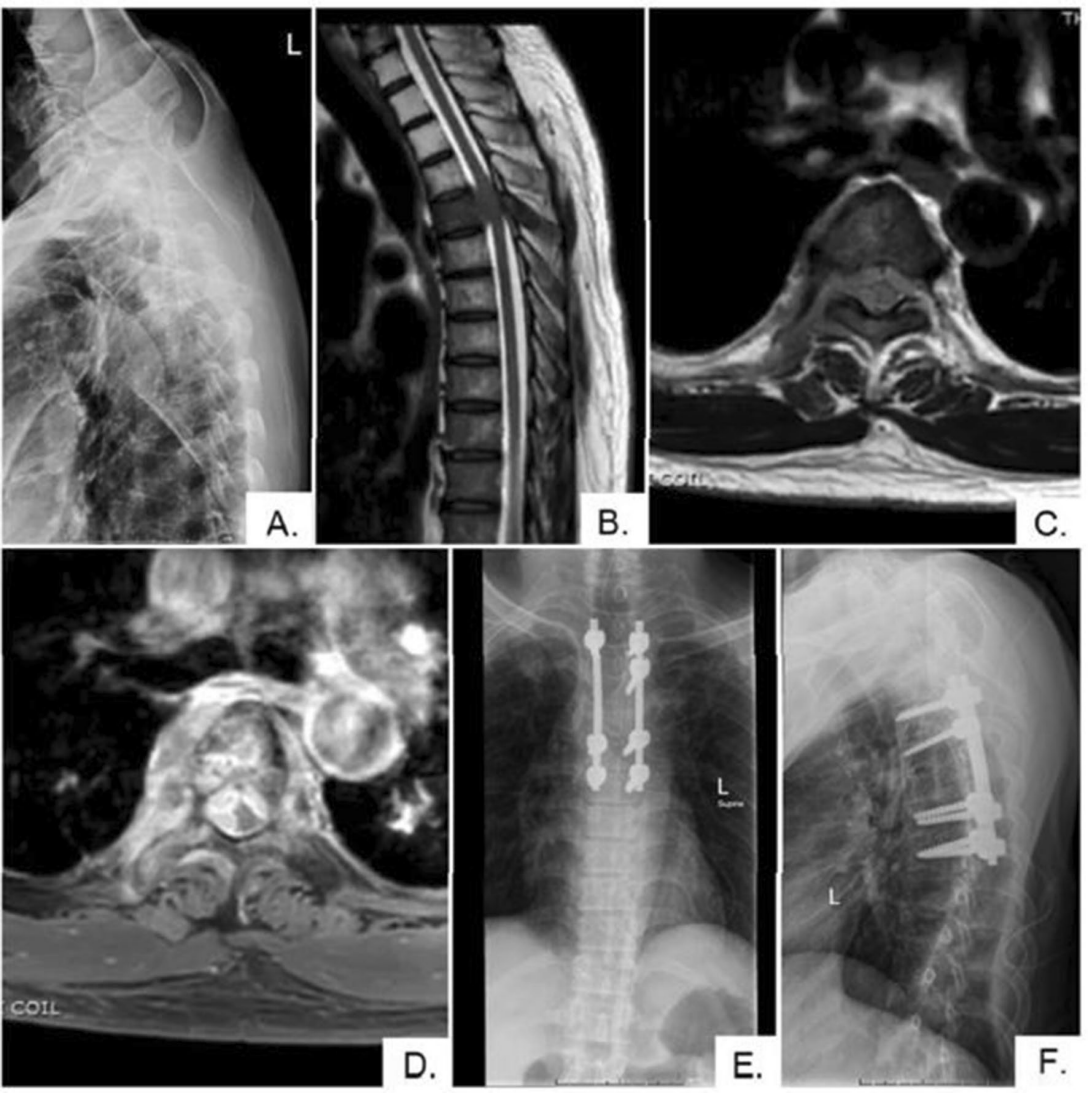
A 57-year-old male patient with tonsil cancer (case No. 14). (**A**, **B**) Lateral plain radiograph and sagittal T2-weighted magnetic resonance image (MRI) showing the mild collapse of the T5 vertebra and bone marrow involvement in the vertebral body due to metastases—extending posteriorly into the spinal canal compressing the spinal cord. (**C**, **D**) Axial T2-weighted and T1-weighted contrast-enhanced MRI confirm the tumor extension into the spinal canal. (**E**, **F**) Anteroposterior and lateral plain radiograph obtained 3 months after posterior instrumentation and decompression without anterior reconstruction, showing no implant-related complication. Please note that the tumor was already involved at the right T4 pedicle at the time of surgery. Thus, the surgeon was unable to insert the pedicular screw securely.

**Figure 2:**
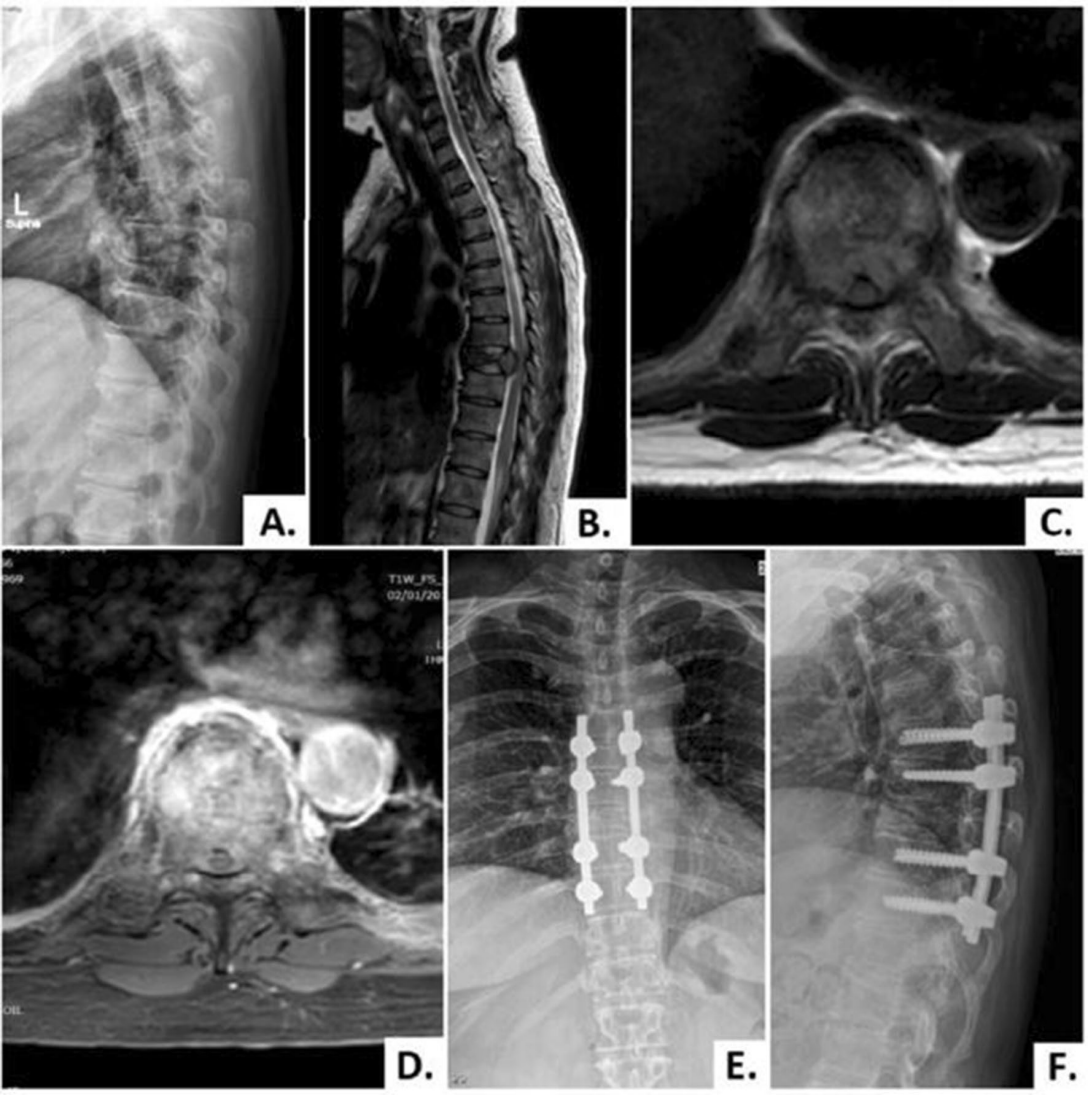
A 49-year-old female patient with breast cancer (case No. 15). (**A**, **B**) Lateral plain radiograph and sagittal T2-weighted MRI showed the collapse of the T8 vertebra and metastases tumor infiltration at the vertebral body with extension posteriorly into the spinal canal. (**C**, **D**) Axial T2-weighted and T1-weighted contrast-enhanced MRI showing enhancement of the vertebral body and both sides of pedicles with the metastatic tumor extension already encased and compressing the spinal cord. (**E**, **F**) Postoperative anteroposterior and lateral plain radiograph obtained at 3-month follow-up visit showing proper implant position without loss of fixation.

All patients were positioned in the prone position on either a Jackson spine table or a radiolucent table with two horizontally placed padded bolsters. A midline incision was then made at the affected region of the spine. Whether a conventional open or percutaneous technique was used, instrumentation with pedicle screws was performed at least two levels above and two below the involved vertebrae. Posterior decompression was then performed via total laminectomy at the involved level and at least one level above and below the involved level. In some cases, partial facetectomy and pedicle resection were also performed to facilitate further resection of the tumor. Example cases that underwent surgery were shown in Fig. 1 and Fig. 2. After surgery, all patients received local radiotherapy and targeted and/or systemic chemotherapy according to our center's multidisciplinary team approach guideline.

### Statistical analysis

Descriptive data in this study is presented as a number, and percentage, mean plus/minus standard deviation, or median and interquartile range. To compensate for multiple comparisons performed at three different points of time, Bonferroni correction was employed. That analysis yielded a statistical significance threshold of *p* < 0.017. Student's *t*-test was used to compare all of the HRQoL assessments between time points. Overall survival was assessed using Kaplan–Meier survival analysis. All statistical analyses were performed using SPSS Statistics version 21.0 (IBM Corporation; SPSS, Inc., Chicago, IL, USA).

## Results

A total of 30 patients (10 males, 20 females) with a mean age of 59.83 ± 11.73 years were enrolled. The lumbar (33.3%) and thoracic (30%) regions were the first and second most involved regions (Table 1). The breast was the most common primary tumor site (40%), followed by lung (26.7%) and prostate (13.3%). Other primary cancer sites included the cervix, colon, liver, renal, and tonsil (3.3% each) (Table 1).

**Table 1:** Demographic and clinical data of 30 metastatic epidural spinal cord compression patients who underwent posterior decompression and stabilization with neither fusion nor anterior reconstruction.

Case no	Gender, age ranges (years)	Primary tumor	Location	Frankel grade		Survival (days)
				Pre*-*operative	Postoperative	
1	F, 60–69	Breast	T3	B	D	1000
2	F, 70–79	Lung	L5	D	D	129
3	F, 60–69	Lung	T5, T6, T11	C	D	325
4	F, 80–89	Bladder	L5	C	C	134
5	F, 40–49	Breast	L1-5	C	C	27
6	F, 50–59	Breast	L4	D	D	65
7	F, 50–59	Breast	L4	C	D	332
8	F, 50–59	Breast	T12, L1, L3–4	D	D	768
9	F, 40–49	Breast	T3–6, L3	C	E	971*
10	M, 50–59	Liver	T4	B	B	198
11	F, 60–69	Lung	T3	C	D	134*
12	M, 50–59	Lung	L3	C	D	572
13	F, 70–79	Lung	T6-9	C	D	647*
14	M, 50–59	Tonsil	T5	C	C	635
15	F, 40–49	Breast	T8	C	D	49*
16	F, 50–59	Breast	T11, L4, L5	C	D	77
17	F, 30–39	Breast	L4–5	D	D	280
18	F, 60–69	Breast	T11, L1, L5	C	D	346
19	F, 60–69	Breast	T12	D	E	379*
20	F, 70–79	Breast	L5	D	D	383*
21	F, 50–59	Cervix	L4	D	D	442*
22	M, 50–59	Colon	L2	D	D	417
23	M, 40–49	Lung	T1–2	B	B	40
24	M, 60–69	Lung	T10	D	E	498*
25	F, 60–69	Lung	L4–5	C	D	631*
26	M, 60–69	Prostate	L4-5	D	E	77
27	M, 70–79	Prostate	T3, T4, T7–8	C	C	118
28	M, 70–79	Prostate	L2	D	E	344*
29	M, 60–69	Prostate	T3	C	D	351*
30	F, 60–69	Renal	L2, L4	C	C	36

*Patient was still alive at the time of the most recent contact.

Regarding pre-operative neurological status, Frankel grade C was found in 53.33% of the patients, followed by 36.67% for grade D and 10% for grade B. No patients were preoperatively rated grade A or grade E. Just over one-half of patients (53.33%) experienced an improvement of at least one Frankel grade during the early postoperative period (Table 2).

**Table 2:** Frankel's grade was compared between the pre-operative and postoperative periods.

Pre-operative grading	Postoperative grading				
	A	B	C	D	E
A	–	–	–	–	–
B	–	2	–	1	–
C	–	–	5	10	1
D	–	–	–	7	4
E	–	–	–	–	–

The pre-operative pain VAS was 57.83 ± 22.84, and that value was improved after surgery to 26.25 ± 21.67 at 3 months (*p* < 0.001), to 25.5 ± 26.29 at 6 months, and 11.67 ± 9.83 at 12 months. The mean pre-operative ODI was 65.35 ± 17.09, and that parameter was significantly improved postoperatively to 48.33 ± 19.69 at 3 months (*p* < 0.001), to 37.27 ± 24.46 at 6 months (*p* = 0.006), and 28.59 ± 15.0 at 12 months (*p* = 0.002). The mean pre-operative EQ5D5L utility score was 0.40 ± 0.33, with postoperative improvement to 0.66 ± 0.32 at 3 months (*p* < 0.001), to 0.75 ± 0.36 at 6 months, and 0.92 ± 0.07 at 12 months (*p* = 0.014) (Table 3).

**Table 3:** Patient health-related quality-of-life (HRQoL) scores compared between preoperation and each follow-up visit until 1 year after surgery.

HRQoL score	Mean ± SD	*P* value
***Pain VAS (0–100)***		
Preoperative (n = 30)	57.83 ± 22.84	
3 months (n = 28)	26.25 ± 21.67	< 0.001*
6 months (n = 10)	25.5 ± 26.29	0.019
1 year (n = 6)	11.67 ± 9.83	0.03
***ODI (0–100)***		
Preoperative (n = 30)	65.35 ± 17.09	
3 months (n = 28)	48.33 ± 19.69	< 0.001*
6 months (n = 10)	37.27 ± 24.46	0.006*
1 year (n = 6)	28.59 ± 15.08	0.002*
***EQ5D5L utility score (− 1 to 1)***		
Preoperative (n = 30)	0.40 ± 0.33	
3 months (n = 28)	0.66 ± 0.32	< 0.001*
6 months (n = 10)	0.75 ± 0.36	0.116
1 year (n = 6)	0.92 ± 0.07	0.014*

*Indicates a statistically significant difference (*p* < 0.017) (alpha level was adjusted using Bonferroni method).

VAS, visual analog scale; ODI, Oswestry Disability Index; EQ5D5L utility score, EuroQOL five-dimension five-level utility score.

The average estimated blood loss was 750.67 ± 529.20 ml (median: 600, IQR: 813), the average operative time was 208.17 ± 58.41 min (median: 190; IQR: 80), and the average length of hospital stay was 14.4 ± 9.47 days (median: 11.5; IQR: 6.25).

A total of four postoperative complications were reported. Three patients had wound dehiscence and infection, and another patient had a local recurrence with progressive neurological deficit (Table 4). Wound complication onset in those 3 patients occurred during 2 weeks to 2 months postoperatively, and all 3 underwent aggressive wound debridement, resuturing, and antibiotic therapy. The patient with local recurrence underwent an extended decompression revision surgery at 4 months postoperatively, and no intra-operative complication was reported.

**Table 4:** Postoperative complications.

Complication	n
Neurological progression	1*
Wound infection/dehiscence	3
Cerebral spinal fluid leakage	0
Instrumentation failure	0
Local recurrence	1*
Revision surgery	1*
Intraoperative mortality	0

*These complications occurred in the same case.

**Figure 3:**
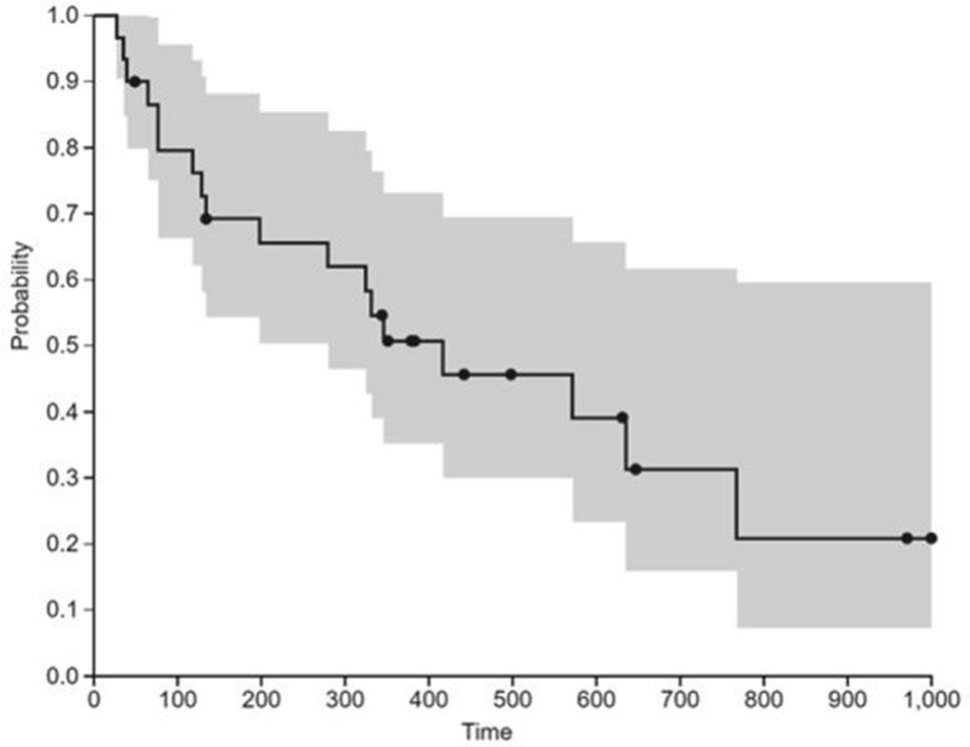
Kaplan–Meier survival curve of the 30 MESCC patients who underwent posterior decompression and stabilization without neither fusion nor anterior reconstruction surgery (black circle = *censored observations*).

Kaplan–Meier analysis estimated the mean survival duration to be 346.83 ± 273.04 days or 11.4 months (median: 338 days, IQR: 516.50–107.75) (Fig. 3).

## Discussion

Spinal metastasis occurs in about 20% of all patients with cancer^12,13^, and about 5–10% develop spinal cord compression^14,15^. Generally, the treatment for spinal metastasis is palliative, aiming to achieve pain relief, maintenance or recovery of neurological function, local durable tumor control, spinal stability, and improved quality of life. The current treatment strategies of spinal metastases included stereotactic spinal radiosurgery (SRS)^16^, chemotherapy, and/or surgery. The purposes are pain relief and control of paralysis—maintaining their performance status^17^. However, in patients with epidural spinal cord compression and/or spinal instability, palliative surgery may be considered as optimal treatment. Lee et al*.*^18^ aimed to predict the vertebral compression fractures (VCFS) after the use of the spinal instability neoplastic score (SINS) prior to SRS in a metastatic spine. They found that patients with higher SINS were more likely to experience symptomatic fractures (31.6%) than those with lower SINS (7.4%). Furthermore, high SINS was a significant risk factor for VCFs after this non-surgical treatment on univariate and multivariate analysis. Therefore, this could also raise the importance of surgical treatment as a valid option in our patients' who mostly present with high SINS, which means spinal instability.

Palliative surgery is one of the treatment modalities of choice in selected patients, intending to achieve adequate spinal cord decompression and stabilization of the metastatic spine^19^. The posterior approach is still the most popular option in the thoracic and lumbar spinal regions due to its simplicity and surgeon familiarity^2^. Several authors have suggested adding anterior reconstruction and/or fusion procedures to achieve even more circumferential decompression and stability. In 2004, Lewandrowski et al.^5^ reported a case series of 30 patients with primary spinal bone tumor or metastasis treated by anterior vertebral reconstruction with fresh-frozen cortical bone allograft with anterior and posterior instrumentation. Despite the high rate of intraoperative or postoperative complications (up to 46%), the authors proposed the anterior column reconstruction technique as being a reliable technique for treating patients with spinal tumors. Jandial et al.^8^ conducted a retrospective case review of 11 patients with spinal metastasis who underwent single-stage posterior-only vertebral column resection and reconstruction with an expandable cage and pedicle screw fixation. They concluded that their approach was viable, and the use of an expandable cage might reduce morbidity associated with extensive mobilization of the nerve roots. Recently, Gezercan et al.^9^ reported a case series of 22 patients who underwent a single-stage posterolateral transpedicular approach with 360-degree stabilization and vertebrectomy in patients with primary or metastatic tumors of the spine. They found the pain VAS and Frankel scores significantly improved postoperatively, and they concluded that their technique is less risky, relatively safe, and less invasive.

Although some studies reported good to excellent results, as mentioned above, others found the add-on procedure(s) to be associated with a higher complication rate, prolonged operative time, and more blood loss. Cahill et al.^10^ conducted a review of 43 cases in 1999. Even though all of their patients remained pain-free until days before they died, they found the use of near-total vertebrectomy followed by anterior and posterior reconstruction to be limited in those with a limited life expectancy. Chen et al.^11^ retrospectively reviewed the surgical results of 23 patients with symptomatic metastatic spinal cord compression at the thoracic spine. They reported comparable outcomes after palliative surgery without anterior vertebral reconstruction, and they concluded that reconstruction might be unnecessary. A recent systematic review by Altaf et al.^20^ found that even though some studies support anterior reconstruction, all of those studies had low to very low evidence quality. Taken together, the aforementioned evidence seems to suggest that the shorter life expectancy and poorer prognosis in this patient population warrant the use of less invasive procedures (such as posterior decompression and instrumentation alone, as described in this study) that yield favorable outcomes and that improve patient quality-of-life.

In general, neurological functions and ambulatory status are significant factors that affect overall patient quality-of-life in metastatic epidural spinal cord compression (MESCC) patients^21^. In our study, 16 of 30 patients (53.3%) had at least one level of Frankel grade improvement within the early postoperative period. However, most patients returned to Frankel D grade, which is characterized by some motor power loss, but they were still able to ambulate with or without gait aid. Several studies (with or without anterior reconstruction and/or fusion) reported similar results. A recent study by Itshayek et al.^22^ reported that 20 of 34 elderly MESCC patients (58.8%) had at least one-grade improvement in their American Spinal Injury Classification (ASIA) impairment scale score after surgery. Similarly, Ju DG et al.^21^ reported that 5 of 9 pre-operative non-ambulatory patients (56%) regained ambulation within one year after surgery. Furthermore, Chang et al.^24^ described improvement of at least one Frankel grade in 22 of 29 MESCC patients (75.9%) who underwent palliative surgery without anterior vertebral reconstruction in the lower thoracic or thoracolumbar region.

In our study, the evaluated surgical strategy was found to be relatively safe. The average estimated blood loss and operative time were 750 ml and 208.17 min, respectively. Even though 13% of our patients had surgery-related complications (three wound complications and one local recurrence with progressive neurological deficit who eventually underwent revision surgery), no cerebrospinal fluid (CSF) leakage, instrumentation failure, or intraoperative mortality was observed. These findings are comparable to those reported from previous studies. Chen et al.^11^ reported a surgery-related complication rate of 17% (one postoperative hematoma, one neurologic deterioration, one CSF leakage, and one wound infection) following palliative surgery without anterior reconstruction and fusion. Similarly, another study^24^ reported a surgery-related complication rate of 6.8% (one CSF leakage and one local recurrence).

Improvement in patient quality-of-life is one of the primary goals of spinal metastasis palliative treatment. In the present study, we found that all HRQoL parameters, including pain VAS, ODI, and EQ5D5L utility-scale, significantly improved at a minimum of 3 months after surgery. There are few reports of outcomes of MESCC patients following palliative surgery. One prospective study of a cohort of 922 patients with spinal metastasis^25^ who underwent surgery found the EQ-5D, pain VAS, and Karnofsky Performance Scale (KPS) index all to be rapidly improved after surgery, and those results were sustained for up to 2 years after surgery among those patients who survived. That group concluded that surgical treatment in this patient population produces rapid pain relief, maintains ambulation, and improves patient quality of life. Similarly, another study^23^ that included pain control usage data and compared KPS scores between before and after surgery found that the median analgesic and steroid used to be significantly lower at 3 months and 6 months after surgery. Interestingly, multivariate analysis in that study revealed a better pre-operative KPS to be an independent predictor of survival in their study population.

## Limitations

The primary limitation of this study is its retrospective design, which rendered it vulnerable to missing or incomplete data in some cases. Another notable limitation is that our limited study population was recruited from single centers. This could limit the generalizability of our findings to another care setting, and the small sample size may have impeded our ability to statistically reveal all existing differences and associations between the two surgical techniques. The last, the dropout rate in this study was high due to the relatively short survival duration of patients with MESCC. That acknowledged, the mean survival in our study was about 11.4 months, which is comparable to the mean survival duration reported by the previous studies^2,11,22–27^.

## Conclusion

Less invasive non-fusion without anterior reconstruction in MESCC patients yields a good result with a low complication rate, improvement in neurological function, and better HRQoL outcome scores. This surgical technique may be considered as a preferable choice of treatment in these groups of patients. However, A prospective multi-center study with a more extended follow-up period is warranted to confirm this study's findings and identify additional information that may enhance surgical approach-related decision-making in this patient population.
